# Understanding Carrier Performance in Low-Dose Dry Powder Inhalation: An In Vitro–In Silico Approach

**DOI:** 10.3390/pharmaceutics13030297

**Published:** 2021-02-24

**Authors:** Joana T. Pinto, Inês Cachola, João F. Pinto, Amrit Paudel

**Affiliations:** 1 Research Center Pharmaceutical Engineering GmbH, Inffeldgasse 13, 8010 Graz, Austria; ines.cachola@gmail.com; 2 iMed.ULisboa–Research Institute for Medicines, Faculdade de Farmácia, Universidade de Lisboa, Av. Prof. Gama Pinto, 1649-003 Lisboa, Portugal; jfpinto@ff.ulisboa.pt; 3 Institute of Process and Particle Engineering, Graz University of Technology, Inffeldgasse 13, 8010 Graz, Austria

**Keywords:** dry powder inhalation (DPI), lactose, carrier properties, physiologically based pharmacokinetic (PBPK) model, powder flow, salbutamol sulphate

## Abstract

The use of physiologically based pharmacokinetic (PBPK) models to support drug product development has become increasingly popular. The in vitro characterization of the materials of the formulation provides valuable descriptors for the in silico prediction of the drug’s pharmacokinetic profile. Thus, the application of an in vitro–in silico framework can be decisive towards the prediction of the in vivo performance of a new medicine. By applying such an approach, this work aimed to derive mechanistic based insights into the potential impact of carrier particles and powder bulk properties on the in vivo performance of a lactose-based dry powder inhaler (DPI). For this, a PBPK model was developed using salbutamol sulphate (SS) as a model drug and the in vitro performance of its low-dose blends (2% *w*/*w*) with different types of lactose particles was investigated using different DPI types (capsule versus reservoir) at distinct airflows. Likewise, the influence of various carrier’s particle and bulk properties, device type and airflow were investigated in silico. Results showed that for the capsule-based device, low-dose blends of SS had a better performance, when smaller carrier particles (Dv_0.5_ ≈ 50 μm) with about 10% of fines were used. This resulted in a better predicted bioavailability of the drug for all the tested airflows. For the reservoir type DPI, the mean particle size (Dv_0.5_) was identified as the critical parameter impacting performance. Shear cell and air permeability or compressibility measurements, particle size distribution by pressure titration and the tensile strength of the selected lactose carrier powders were found useful to generate descriptors that could anticipate the potential in vivo performance of the tested DPI blends.

## 1. Introduction

Dry powder inhalers (DPIs) are dosage forms used to deliver drugs to the lung. It is generally accepted that the active pharmaceutical ingredient (API) particles must have a size between 1 and 5 µm to enter the respiratory tract and deposit on the bronchio-alveolar epithelium [1]. Due to their large specific surface area, inhalable drug particles are very cohesive, present poor flowability and are a challenge to process in order to be efficiently delivered to the lung. To improve their flowability, the inhalable API powders are often blended with larger excipient particles, through a practice known as adhesive mixing [2]. Adhesive mixing aims to promote the attachment of the API to the surface of coarser free flow excipient particles, so that the drug can be “carried” more easily with improved handling. Therefore, the coarse excipient particles are commonly known as carriers. While the API particles are expected to attach strongly enough to the carrier surface to avoid problems such as poor flowability and segregation, the adhesion should not be so strong that would prevent aerosolization of the API particles (that need to be detached from the large excipient particles to reach their therapeutic targets in the lung) [3]. Considering that most of the DPI inhalers rely on the inspiratory airflow of the patient to fluidize the powder and promote the API particle detachment from the carrier and release from the device, a strong interdependency arising from formulation, device type and patient related factors is present.

Particles of α-lactose monohydrate are the most common DPI carriers. Lactose intended for inhalation is commercially available in many grades (e.g., particles with different shapes, roughness, size distribution) [4]. Depending on the intended application for the DPI, distinct carrier characteristics might be necessary. For instance, it is known that carriers with different sizes and surface roughness can result in distinct forces involved in API attachment-detachment, influencing fluidization efficiency [5,6,7]. Fluidization efficiency will also depend upon the type of device used and its internal airflow resistance [8]. High-resistance inhaler devices are known to be more effective in using the kinetic energy derived from the airflow stream of the patient resulting in greater deposition [9]. It is also well-known that the presence of a certain percentage of fine excipient particles can benefit the aerosolization performance through various mechanisms (i.e., shift of the fluidization mechanism, impact on the surface topography of coarse carrier particles, agglomeration with the API) [10,11]. Additionally, an interdependency between fine content and the type of device has also been demonstrated [12]. Consequently, it is important to distinguish and select amongst the different available lactose grades, those that have the necessary particle characteristics to achieve the desired performance using the intended device in the target patient population. Physiologically based pharmacokinetic (PBPK) modelling can play a pivotal role in identifying accurately the optimal particle–device combination to achieve the aforementioned purpose for a given therapeutic use.

Contrary to conventional pharmacokinetic (PK) models that are mainly defined by drug related data, PBPK models are established using the anatomical and physiological structure of the species studied [13]. Consequently, a PBPK model considers the clinically relevant factors of formulation and patient providing the mechanistic basis to describe the uptake and disposition of a given API by the extrapolation of its PK profile to its in vivo performance, depending on its dose, route and target species response [13]. Therefore, PBPK models are used to simulate the pharmacokinetic profiles of the API at various physiological conditions. Besides the physiological and biological parameters of the targeted species, drug-related information is utilized to construct a PBPK model. Drug-related information includes specific PK disposition parameters (e.g., clearance, blood to plasma ratio, volume of distribution), molecular properties (e.g., molecular weight, pKa, LogP, equilibrium solubility) and physical characteristics of the DPI (e.g., dosage form, particle size distribution, dose, apparent solubility). Recently, regulatory authorities have encouraged the use of PBPK models to support decision making during the life cycle of drug products [14,15]. In this context, the use of the data generated in vitro during the characterization of the DPI can provide important input parameters for the prediction of the in vivo pharmacokinetic profiles of the API using PBPK models and anticipate, in silico, the performance space intended for the product.

A large number of reports have shown the importance of characterizing distinct particle properties while attempting to anticipate the aerosolization performance of carrier based DPIs [11,16,17]. For example, different carrier particles porosities (nanoporosity versus macroporosity) have been associated with distinct impacts on fluidization performance. The pores smaller than the API particle size are shown to be advantageous than larger ones [18,19]. In the case of the carrier particle shape, elongated particles are suggested to be more efficient in terms of lung deposition of the API [20,21]. In contrast, other works suggest these might be only the case because, smaller, more elongated carriers are generally smoother [22]. Therefore, the effect of one property over the other on the performance of DPI is difficult to isolate as many of the surface and particle level properties are associated with one another. Alternatively, bulk powder properties such as flowability have also been used in an attempt to anticipate aerosolization efficiency. The extent of air permeability of the powder bed has been used as an indicator of fluidization efficiency during oral inhalation. Here, the powders with a higher fraction of fines were less permeable to air, resulting in a better API deposition [12,18,23]. Moreover, different cohesion propensities have been associated with distinct fluidization mechanisms [23]. In contrast, other works have found no correlation between fluidization and powder flowability measurements [24]. Direct estimation of the API particles detachment from the carrier particle has also been applied in order to try to predict aerosolization performance. One of these techniques measures particle size distribution (PSD) by pressure titration. In this, the PSD of an API and carrier blend is monitored using increasing pressures to disperse the powder until a stable PSD is attained. When no further changes in PSD are observed, it is assumed that the API particles have been detached from the carrier, allowing the comparison of different DPI blends by the pressures needed to disperse them [25].

In this work, the different methodologies (i.e., characterization of particle properties, powder bulk flowability and API detachment) were explored in order to characterize the attributes of four DPI formulations containing different grades of lactose particles and the model drug salbutamol sulphate (SS). Based on this and using an in vitro–in silico approach, it was deemed paramount to understand the potential impact of particle and bulk properties of the selected carriers on the in vivo performance of DPI products. To this end, the in vitro aerodynamic performance of SS in low dose blends with the selected carrier particles was firstly characterized using different powder inhalation scenarios, i.e., distinct inhaler types (capsule versus reservoir) and increasing airflow rates. Thereafter, a PBPK model for SS was developed, and the influence of various particle and bulk properties on different in vivo performance descriptors (derived in silico) was statistically categorized.

## 2. Materials and Methods

### 2.1. Materials 

Micronized salbutamol sulphate (racemic mixture, Fagron GmbH & Co., Glinde, Germany) with a particle size of Dv_0.1_ = 0.43 ± 0.01 μm, Dv_0.5_ = 1.53 ± 0.07 μm and Dv_0.9_ = 3.79 ± 0.26 μm was chosen as a model API. In line with our previous work [1], Duralac^©^ H (16% α anomer and 83.5% β anomer, Meggle, Wasserburg am Inn, Germany), α-lactose monohydrate Flowlac^©^ 90 (β anomer ≤ 3%, Meggle, Wasserburg am Inn, Germany), Respitose^©^ SV003 (β anomer ≤ 3%, DFE pharma, Goch, Germany) and Lactohale^©^ 100 (β anomer ≤ 3%, DFE pharma, Goch, Germany), were used as model carriers. Flowlac^®^ 90 was dry sieved using a vibratory sieve shaker (Retsch AS200, Germany) to obtain its 20–90 μm particle size fraction. Purified water (TKA Wasseraufbereitunssystem GmbH, Niederelbert, Germany) and acetic acid (Emprove, Merck Millipore, Burlighton, MA, USA) were used to dissolve SS.

### 2.2. Characterization of Particle Properties

#### 2.2.1. Gas Adsorption

Before gas adsorption measurements (*n* = 2) were carried out in a Tristar II 3020 (Micromeritics, Norcross, GA, USA), the samples were dried under vacuum (0.15 mbar) overnight at 30 °C (VacPrep 061, Micromeritics, Norcross, GA, USA). The samples were analyzed at 77.350 K using a nitrogen relative pressure (p/p_0_) in the 0.01–0.99 range. The specific surface area of the powders was calculated using the Brunauer, Emmmett and Teller (BET) adsorption theory (7-point analysis in the 0.05–0.2 p/p_0_ range). To determine the macro- and mesopores size distribution, a modification to the Kelvin equation (Equation (1)) was used (Barrett–Joyner–Halenda method) [2].

(1) $$\log\left(\frac{P}{P_{0}}\right)=\frac{2\sigma V}{{\mathrm{RTr}}_{k}}$$

where σ and V are the surface tension and molar volume of liquid nitrogen, respectively, R is the universal gas constant, T the absolute temperature and r_k_ the Kelvin radius. The radius of the pores at the different relative pressures follows directly from the sum of the thickness of adsorbed nitrogen layer with the r_k_.

#### 2.2.2. Helium Pycnometry

The particle density was calculated as the ratio of the sample mass by its volume (*n* = 2). The volume of the powders was determined by helium pycnometry using an AccuPyc II 1340 (Micromeritics, Norcross, GA, USA). Before measurement, the powders were accurately weighted and their volume determined using 20 gas purges at 19.5 psi and an equilibrium rate of 0.005 psi/min during five consecutive runs.

#### 2.2.3. Surface Morphology and Shape Coefficient

The morphology of the distinct lactose particles was qualitatively evaluated at different magnifications using scanning electron microscopy (SEM). To be observed under the SEM microscope (Zeiss Ultra 55, Zeiss, Germany) operating at 5 kV, the powders had to be sputtered with gold-palladium prior to the measurement.

The shape coefficient (α) of the different lactose particles was calculated according to Wong and Pipel (Equation (2)) [3]:

(2) $$\alpha \;=\;\mathrm{SSA}\rho_{s}d_{e}\;+\;N$$

where SSA is the specific surface area determined by gas adsorption, *ρ_s_* the particle true density, N the elongation ratio (the ratio of the particle length by its width), and d_e_, the Heywood equivalent diameter, was calculated according to Equation (3):

(3) $$d_{e}={\left(\frac{3.08\mathrm{WL}}{\pi }\right)}^{\frac{1}{2}}$$

where W and L are the particle width and length, respectively. The width and length of the particles of 60 to 200 particles were determined from SEM images using ImageJ software (National Institutes of Health, Gaithersburg, MD, USA). The maximum and minimum Ferret diameters were considered to be the particle length and width, respectively [4].

### 2.3. Blending 

Adhesive mixtures of 2% (*w*/*w*) SS were prepared. Lactose (49 g) and 1 g of API were weighed into stainless steel vessels using a sandwich methodology. For the sandwich, the API was placed between two even layers of lactose, and the half-filled vessels blended in a Turbula blender TC2 (Willy A. Bachofen Maschinenfabrik, Muttenz, Switzerland) for 90 min at 62 rpm. The homogeneity of the blends was confirmed as previously described [1,5], and characterization of the mixtures was only carried out once a homogenous sample was obtained.

### 2.4. Characterization of Powder Bulk Properties

#### 2.4.1. Laser Diffraction by Pressure Titration 

The particle size distribution of the raw materials and respective blends was evaluated using laser diffraction (HELOS/KR, Sympatec GmbH, Clausthal-Zellerfeld, Germany). The powders were placed on a vibrating chute (Vibri, Sympatec GmbH, Clausthal-Zellerfeld, Germany) and dispersed using a dry dispersing system (RODOS, Sympatec GmbH, Clausthal-Zellerfeld, Germany). The powders were sampled during 10 s and the measurements with an R2 (0.45–87.5 μm) and R5 lens (4.5–875 μm) triggered once an optical concentration (C_opt_) of 0.5% was reached. To evaluate the pressure at which the particles de-agglomerate, pressure titration was applied. For this, the primary dispersion pressure (PDP) was manually adjusted in 0.2 bar steps in the range of 0.1–2.0 bar. Measurements were done in triplicate (*n* = 3) at 0.1, 1.5 and 2.0 bar. Before each measurement, the dispersing system was cleaned using sand and a reference measurement was taken. The resulting volumetric particle size distributions were calculated and analyzed using Windox 5 software (Sympatec GmbH, Clausthal-Zellerfeld, Germany).

#### 2.4.2. Hardness and Tensile Strength 

Plugs of lactose and their blends were prepared using a hydraulic press applying a load of 500 Kg and the thickness of the compressed powders kept constant (3.69 ± 0.27 mm) as well as their diameter (10 mm). The hardness of the resulting plugs was evaluated using a 3-in-1 hardness, diameter and thickness testing instrument (PTB 311E, PharmaTest GmbH, Hainburg, Germany). Measurements were done in quintupled (*n* = 5) and the tensile strength calculated using Equation (4), where P is the applied load and D and t the diameter and thickness of the compacts, respectively [6].

(4) $$\mathrm{Tensile}\;\mathrm{Strength}\;=\;\frac{2P}{\pi \mathrm{Dt}}$$

#### 2.4.3. Dynamic Powder Flow Analysis

To measure the flow properties of the samples, a FT4 Powder Rheometer (Freeman Technology, Tewkesbury, UK) was used. Powder compressibility, permeability and cohesivity were evaluated using a borosilicate glass cylinder with an inner diameter of 25 mm. To remove the packing history of the powder and operator’s influence, all samples were conditioned using a 23.5 mm blade which was moved down in a helicoidal path, allowing the displacement of the powder and generating a uniform low packing powder bed. To remove the surplus powder, the cell was split, and the blade was replaced by a vented compaction piston. To evaluate the compressibility of the powders (*n* = 3), their bulk density was obtained at eight different points by compressing the sample at a varying normal stress range between 1 and 15 kPa. For air permeability (*n* = 3), the pressure drop (ΔP) across the powder bed was measured at a constant airflow velocity of 2mm/s, in the varying normal stress range of 1 to 15 kPa (eight ΔP values were taken). For powder cohesivity, an appropriate amount of sample was placed in a 1 mL cell in order to fill it, and after the conditioning cycle, the surplus of powder was removed and the blade replaced by a 24 mm shear cell. The powder was pre-sheared at 15 kPa and then sheared at 9, 8, 7, 6, and 5 kPa. The flowability parameters of the powders were obtained from the Mohr’s stress circles.

### 2.5. Evaluation of the Aerodynamic Performance 

The aerodynamic performance of the blends was determined using a low-resistance capsule-based inhaler (Cyclohaler^®^, Ratiopharm, Vienna, Austria), with a pressure drop of 4.0 kPa at airflows higher than 100 L/min. Additionally, a multi-dose (reservoir) high to medium resistance device was also used; this presented a pressure drop of 4.0 kPa at 55 L/min. Considering, the known sensitivity of the capsule-device to different airflows, the next generation impactor (NGI, Copley Scientific, Colwick, Nottingham, UK) tests were performed at 28, 60 and 100 L/min using the capsule inhaler. Whereas for the reservoir device, the NGI test was performed only at 60 L/min. The airflows were adjusted with an accuracy of ±2.0% using a critical flow controller (TPK, Copley Scientific, Colwick, Nottingham, UK) and a flow meter (DMF4 model, Copley Scientific, Colwick, Nottingham, UK) operating according to the differential pressure principle. For the capsule-based device, about 40 mg of the blends were filled into four gelatin capsules (size 3, Capsugel, Bornem, Belgium) that were discharged into the NGI. For the reservoir device, 10 consecutive shots were fired into the NGI and analyzed. For all the experiments, 4.0 L air were made to pass-through the impactor in order to discharge the inhalers. Each experiment was carried in triplicate and the SS recovered from the NGI stages, pre-separator and induction port quantified via high performance liquid chromatography (HPLC) [5]. Briefly, an Agilent HPLC, equipped with a photodiode array detector (Waters, Milford, MA, USA) was used to quantify salbutamol at 276 nm. The HPLC was carried out in a Phenomenex Luna 5 µm, C18 100Å, 150 × 4.6 mm using an isocratic method at a flow rate of 1 mL/min, a column temperature of 30 °C and a sample temperature of 20 °C. The mobile phase consisted of 60% aqueous solution containing 0.1% hexane sulfonic acid and 1% (*v*/*v*) acetic acid mixed with 40% MeOH. The method was confirmed to be linear in the 0.50 µg/mL to 200 µg/mL range. Following HPLC quantification the fine particle mass (FPM) was considered as the mass of particles with an aerodynamic size between 1 and 5 µm and the fine particle fraction (FPF) was estimated as the ratio of the FPM mass to the emitted dose (ED). The median mass aerodynamic diameter (MMAD) and its geometric standard deviation (GSD) calculated based on the Log-Normal distribution of the particles’ sizes.

### 2.6. PBPK Modelling of Inhaled Salbutamol Sulphate 

#### 2.6.1. Determination of Salbutamol Sulphate Solubility 

The apparent solubility of crystalline SS (*n* = 3) was determined in phosphate buffer (PBS, pH 7.4). An excess amount of SS was added into a 5 mL of PBS and the suspension maintained at 37 °C, 100 rpm for 24 h under constant agitation. The resulting supernatant was withdrawn and filtered using mixed cellulose ester membrane filters presenting a mesh size of 0.45 µm. The withdrawn sample solution was diluted using PBS buffer and analyzed by HPLC [5] to quantify the concentration of SS, thus its solubility.

#### 2.6.2. Model Development and Validation

Based on the in vivo plasma concentration profiles reported by Moore et al., after inhalation of 600 µg of salbutamol powder from a Diskus^®^ device (in presence of charcoal) [7], a PBPK model was developed in GastroPlus^®^ 9.6 (GP, Simulations Plus, Lancaster, CA, USA) using its Integrated Pulmonary Compartmental Absorption and Transit^®^ module (PCAT). The deposition of SS was predicted in silico using a 1-D computational stochastic lung model (with 60th percentile of the airways) applied in the Multiple-Path Particle Dosimetry v2.11 software (MPPD, Applied Research Associates, Inc, Albuquerque, NM, USA). The in vitro deposition profiles of SS from Diskus^®^ at 90 L/min were extracted from literature and used to calculate the MMAD and the GSD of the deposited drug [8]. The MMAD and the GSD were inserted into MPPD and, in combination with spirometry profiles of Diskus^®^ [9], applied to calculate the deposition of SS using an Oronaso-Augmenters model in upright subjects. The deposition profiles were added into the PCAT model within GP and used to predict SS plasma concentration over time in 30-year-old healthy male subjects with an average weight of 77 kg and a body-mass-index (BMI) of 24.9 kg/m^2^ [7]. The physicochemical parameters of SS were calculated using GP ADMET Predictor^®^ and used for the PPBPK simulations, except the solubility at pH = 7.4 and its density that were experimentally determined. The mean particle size and respective deviation were assumed to be the MMAD and GSD, respectively. All the tissues in the PBPK model were assumed to be perfusion limited and their tissue to plasma partition coefficients calculated using the Lukacova method [10]. Additional parameters necessary for the construction of the PBPK model and their respective sources are resumed in Table 1. Lastly, considering that the observed plasma concentration profiles of SS were acquired with the concomitant delivery of charcoal, the first pass extraction was considered to be 100%. No transporters or enzymes were specified.

#### 2.6.3. In Silico PK Prediction of Salbutamol Sulphate Delivery to the Lung Using Distinct Carriers

The developed model for SS was further used for the in silico prediction of its delivery from the adhesive blends with the distinct carriers using the different devices and flow rates. For this, the in vitro aerodynamic profiles obtained under various circumstances were used as inputs for MPPD and the deposition in the lung calculated in silico using the same approach described in the development of the model. The inhalation volume was assumed to be 4.0 L and the time adjusted (i.e., 8.6, 4.0 and 2.4 s) according to the different tested airflows (i.e., 28, 60 and 100 L/min). Similarly, to Diskus^®^ an exhalation to breath-hold ratio of one was assumed, and the breath-hold was considered to be 10 s [15]. The deposition profiles of SS in combination with the different lactose carriers were obtained from MPPD and input into the inhalation model developed using GP. Likewise, the time dependent plasma concentration profiles and urine excretion of salbutamol could be derived and compared.

### 2.7. Statistical Analysis 

A two-way analysis of variance (ANOVA) without replication was carried out in Excel (Microsoft, Seattle, WA, USA). The Pearson correlation coefficients between particle and bulk level properties of carrier powders and the aerodynamic performance parameters as well as the pharmacokinetic descriptors derived in silico were calculated using OriginPro 2020 (OriginLab, Northampton, MA, USA).

## 3. Results

### 3.1. Carrier Particle Properties 

**Figure 1:**
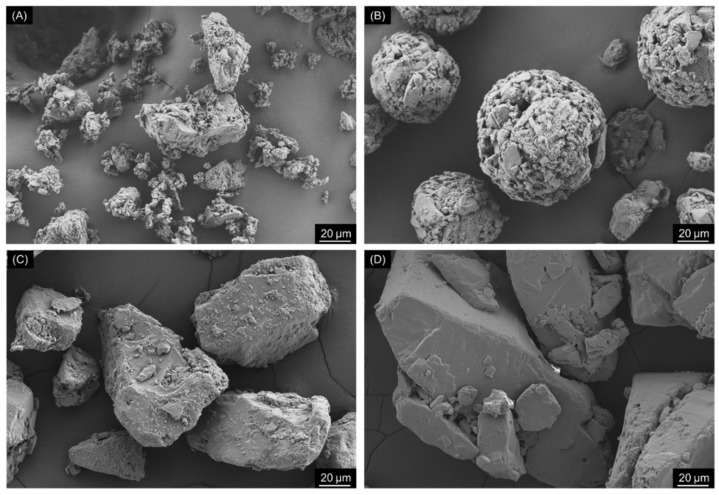
Scanning electron microscopy images of the different grades of lactose selected for this study: (**A**) Lβ, (**B**) LHα-sph, (**C**) LHα-tom-sm and (**D**) LHα-tom-larg.

Various lactose grades presenting distinct solid-states, particle size distribution, shapes and morphologies were selected as carriers. The summary of their characteristics is given in Table 2, and their SEM micrographs are depicted in Figure 1. Duralac^©^ H was chosen due to being predominately composed of anhydrous β-lactose, hereafter referred to as Lβ. This lactose showed to be composed of irregular aggregates, resulting in particles with a relatively large SSA, pore volume and shape coefficient. However, its macro- and meso-pores (as determined by gas adsorption) were small in size. Contrary to the other selected lactose grades, Lβ was also constituted by a large percentage of particles <10 µm.

Flowlac^©^ 90 was composed of spherical agglomerates of α-lactose monohydrate, the polymorph of lactose generally used in the formulation of DPI powders (and is hereby denominated LHα-sph) [16]. The surface of lactose agglomerates of LHα-sph appeared to be very rough and contain deep pores, resulting in relatively larger meso- and macropores. Moreover, this lactose also revealed to have an intermediate SSA and a large pore volume, resulting in an intermediate shape coefficient value. Lastly, this was the carrier with the lower percentage of particles <10 µm.

Finally, two grades of tomahawk α-lactose monohydrate, the carrier particles typically used in DPI, were also evaluated. The two carriers, Respitose^©^ SV003 and Lactohale^©^ 100, were morphological identical but had distinct mean particles sizes and are hereafter called as LHα-tom-sm and LHα-tom-larg, respectively. The tomahawk shaped particles by comparison with the other lactose grades presented a smoother surface and showed the smallest values of SSA, pore volume, pore size and shape coefficient. The LHα-tom-sm, the smaller of the tomahawk grades, presented a mean particle size in the same range of the Lβ and LHα-sph. Compared to its larger counterpart, the LHα-tom-sm showed a larger percentage of particles <10 µm, SSA, pore volume and pore size but a smaller shape coefficient.

### 3.2. Bulk Properties of the Powders 

The bulk properties of the raw materials as well as the ones of their blends with SS were characterized employing PSD by pressure titration, tensile strength determination and measurement of their flowability using powder rheology. Although, tensile strength is a measure usually reserved for products subjected to high pressures, which is not the case of DPI blends, given the exploratory nature of this work, it was decided to include it and explore its usability as a descriptor for API attachment.

#### 3.2.1. Raw Materials

**Figure 2:**
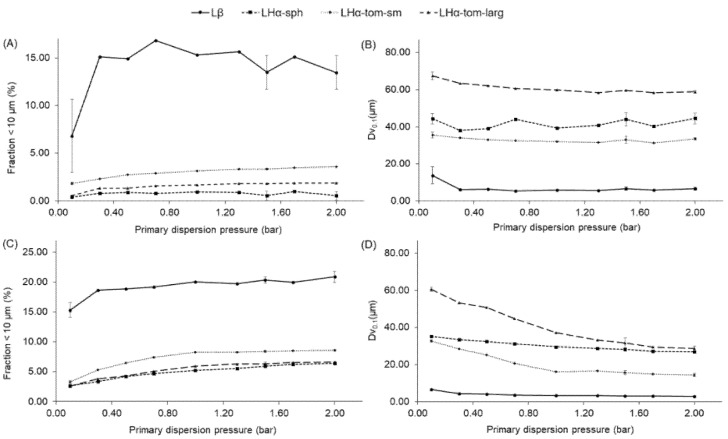
Pressure titration results for the different excipient particles: (**A**) fine fraction and (**B**) Dv_0.1_ as well as their blends with salbutamol sulfate (**C**) fine fraction and (**D**) 10th percentile of the particle size distribution (by volume). Triplicates were carried out at 0.1, 1.5 and 2.0 bar (mean ± SD).

To investigate the dispersion behavior of the particles with the potential to reach the large and small respiratory airways, the fraction <10 µm and the 10th percentile of the particle size distribution by volume(Dv_0.1_) [17] were analyzed by pressure titration. As seen in Figure 2A,B only the PSD of Lβ was influenced by different PDPs. For instance, its fraction of particles <10 µm doubled when increasing the PDP from 0.1 to 0.3 bar, then remained the unchanged. A similar trend was found for the evaluation of the Dv_0.1_ of Lβ across the used PDPs.

**Figure 3:**
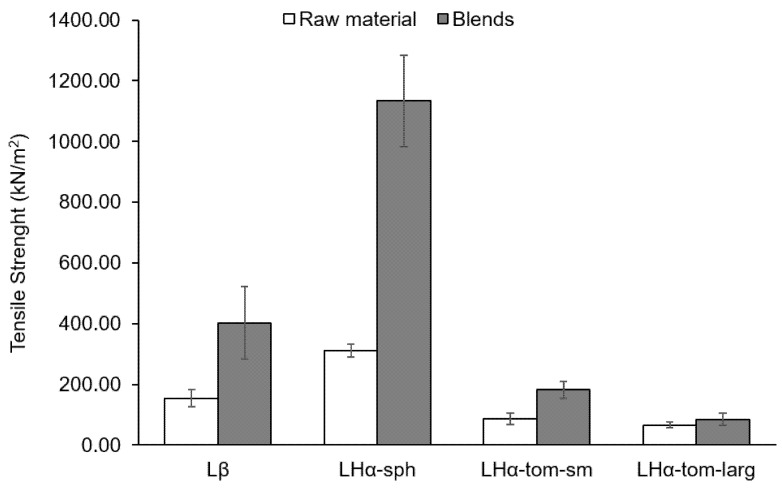
Tensile strength of the different excipient particles and their blends with SS (*n* = 5, mean ± SD).

The tensile strength (TS) was evaluated in order to understand the different compaction abilities of the carriers (Figure 3). It was observed that the LHα-sph presented the highest value of TS, followed by Lβ and LHα-tom-sm. The largest tomahawk carrier revealed the smaller value of TS.

Flowability results for the different lactose grades are summarized in Table 3. It was observed that Lβ presented the highest cohesion (Coh) and lower flow function (FF) values and LHα-sph presented the lowest ones. The two tomahawk carriers (LHα-tom-sm and LHα-tom-larg) presented similar intermediate values of Coh and FF. Concerning the incipient shear stress values (SSi) across the applied normal stresses (5–15 kPa), it was observed that the Lβ presented the highest mean (σ), followed by LHα-tom-sm and LHα-tom-large, that presented similar behaviors. LHα-sph showed the lowest σSSi. The difference (Δ) between the measured SSi at 5 and 15 kPa was larger for Lβ and LHα-tom-sm that presented similar ΔSSi values. LHα-sph presented an intermediate ΔSSi value, and the lowest difference was observed for the LHα-tom-larg. The mean value of change in volume after compression (σCPS) across the applied normal stresses of 1 to 15 kPa was notably larger for Lβ, followed by LHα-tom-sm and LHα-sph. The LHα-tom-larg presented the smallest value. A similar trend (i.e., Lβ > LHα-tom-sm > LHα-sph > LHα-tom-larg) was observed in the difference (Δ) of change in volume after compression (CPS) at 1 and 15 kPa. The mean (σ PD) and difference between 1 and 15 kPa (ΔPD) values of pressure drop across the powder bed also appeared to follow the trend: Lβ > LHα-tom-sm > LHα-sph > LHα-tom-larg.

#### 3.2.2. Powder Blends 

Characterization of the powder blends PSD by pressure titration (Figure 2C,D), revealed that when the samples were tested at 0.3 bar the Lβ + SS blend showed the most notable increase in the <10 µm fraction and decrease in the Dv_0.1_. The <10 µm fraction continued to slowly increase until 1 bar when this seemed to stabilize (Figure S1 in the Supplementary Material). The Dv_0.1_ steadily decreased until a plateau was reached at 1 bar and subtly decreased again from 1.5 to 2.0 bar (potential milling of the crystalline fine powders at the high PDP used [18]). For LHα-sph + SS, the <10 µm fraction and the Dv_0.1_ steadily increased and decreased across the used PDPs, respectively. For the LHα-tom-sm + SS blend, it was observed that the <10 µm fraction and the Dv_0.1_ steadily increased and decreased, respectively, until a plateau was reached at 1.0 bar. The LHα-tom-sm + SS powder also showed a subtle decrease of the Dv_0.1_ between 1.5 and 2.0 bar. Finally, for the LH-tom-larg + SS blend, a similar trend was observed to the one seen for the mixture of SS with LHα-sph. However, in comparison to the LHα-sph + SS blend, the LH-tom-larg + SS powder showed a larger difference in the overall PSD of the mixture, in particular for the Dv_0.1_ fraction (Figure 2D).

The hardness of the mixture’s compacts evidenced that the addition of SS led to larger values of TS (Figure 3), with the exception of the mixture containing LHα-tom-larg + SS. For the latter, the TS values in presence of SS were similar to when the carrier was compacted alone. For the rest of the carriers, the most notable increase in TS was observed for the compacts containing LHα-sph + SS followed by Lβ + SS > LHα-tom-sm + SS.

Concerning the flowability of the mixtures, it was determined that the Coh and FF values were too variable and thus not suitable for proper interpretation (these were excluded and are not shown). We hypothesize that this could have been due to detachment of the API during the measurement and the consequent segregation of the powders (carrier and API), resulting in higher variability in the extrapolation of the major primary and unconfined yield stress as well as cohesion values from the Mohr’s circles of stress. Analysis of the absolute σSSi values (Table 4) shows that in relation to the raw materials the presence of SS resulted in a slight increase of these, with the exception of the mixtures with LHα-sph where a decrease was found. Similar trends to when the carriers were analyzed alone were detected, that is, the σSSi was the highest for Lβ + SS, followed by LHα-tom-sm + SS > LHα-tom-large + SS > LHα-sph + SS. For the absolute values of ΔSSi, a slight increase was also seen for the mixtures with Lβ + SS and LHα-tom-sm + SS and a decrease observed for the one with LHα-sph + SS. In contrast, for LHα-tom-larg + SS, a very notable increase (double the value when the carrier was analyzed alone) in the ΔSSi values was observed. As a consequence, in the case of the mixtures, the sample showing the lowest ΔSSi was the LHα-sph + SS. For the absolute σCPS values, it was also observed that these increased with the addition of SS, with the exception of the mixtures containing LHα-tom-sm + SS, where a decrease was found. The largest difference was observed for the mixtures with Lβ + SS. Overall, compared to when the carriers were tested alone, similar trends were found for the blends with SS, i.e., the σ CPS were the highest for Lβ + SS, followed by LHα-tom-sm + SS > LHα-sph + SS > LHα-tom-large + SS. Moreover, for the ΔCPS, a similar trend to the one found for the raw materials was seen (i.e., Lβ + SS > LHα-tom-sm + SS > LHα-sph + SS > LHα-tom-larg + SS) the addition of the API resulted only in a very slight increase of the absolute ΔCPS values. For the σPD values, a decrease in the absolute values in relation to when the raw materials were tested alone was observed for all the mixtures; this resulted in a similar trend of air permeability as the one observed for the lactose grades alone (i.e., Lβ + SS > LHα-tom-sm + SS > LHα-sph + SS > LHα-tom-larg + SS). In relation to the raw materials, the absolute ΔPD values decreased for the mixtures containing Lβ + SS and LHα-tom-sm + SS, and no difference could be found when SS was mixed Lα-sph and LHα-tom-larg. However, the differences detected did not produce a significant change, and a similar overall trend to the raw materials was observed in respect to the ΔPD values (i.e., Lβ + SS > LHα-tom-sm + SS > LHα-sph + SS > LHα-tom-larg + SS).

### 3.3. Assessment of the In Vitro Aerodynamic Performance 

Relating to the in vitro aerodynamic performance, some differences were observed between the behaviors of the DPI blends containing distinct carriers (Table 5). The different airflows used to test the capsule-based device did not have a notable impact on the emitted dose of SS. However, these had a slight effect on the FPM, FPF and MMAD of the API. In combination with the capsule-based device, the blend containing Lβ showed the highest FPM and FPF values. In addition, among the blends containing different carriers, the SS fine particle values (FPM and FPF) were less affected by the airflow when the API was blended with Lβ. In contrast, LHα-sph behaved poorly as a potential carrier for SS, as it can be seen by the very low values of FPM (≤0.20 mg) and FPF (<6.00%). The FPF and FPM values of LHα-sph were notably affected by the airflow, and at 28 L/min, almost no drug could be found at the lower stages of the NGI. The blends containing LHα-tom-sm also performed fair in the capsule-based device. With this carrier, notable differences in the FPF and FPM fraction were only observed when the airflow decreased from 60 to 28 L/min. The LHα-tom-larg + SS blend seemed to be most affected by the different airflows used to activate the Cyclohaler^®^ as the FPM and FPF values of the API steadily deteriorate from 100 to 28 L/min. However, this was the only carrier to demonstrate comparable performance when used in combination with the capsule and reservoir devices. As shown by the very low ED and FPM values, all the other potential carriers showed a very poor performance when used in combination with the reservoir inhaler. Generally, it was observed that the MMAD increases with a decrease in the airflow rate and that the blends with LHα-sph + SS presented the highest MMAD values followed by the mixtures with: Lβ + SS < LHα-tom-sm + SS < LHα-tom-larg + SS.

### 3.4. In Silico Prediction of Salbutamol Sulphate Deposition Plasma Concentration Profiles from Diskus^®^

**Figure 4:**
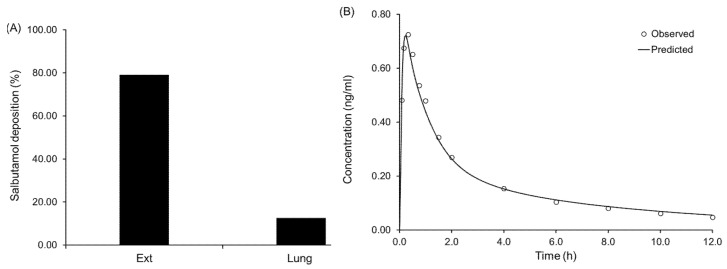
In silico prediction of salbutamol deposition (**A**) and plasma concentration (**B**) after delivery from Diskus^®^. Ext: extra-thoracic.

In line with our previous works, we decided to carry out the in silico predictions of SS deposition using MPPD and couple it with GastroPlus (GP) [19,20]. Given literature information reporting that healthy adult volunteers inhale through Diskus^®^ at a mean flow rate of 98.57 ± 27.86 L/min during 2.8 ± 0.83 s [9], it was found appropriate to use an average of the in vitro aerodynamic profiles of SS (in combination with Diskus^®^) obtained at 90 L/min during 2 and 4 s. The MMAD and its GSD were calculated based on the Log-Normal distribution of the particles’ sizes (Table 1). These values were input into MPPD in combination with the drug true density. An inhalation volume of 4520 mL during 2.8 s was considered in combination with an exhalation of 9.7 s and breath-hold of 10.3 s [9]. All other parameters were left as default, and the lung deposition of SS was calculated to be 12.6%, with 79.2% of the drug remaining in the extra-thoracic (Ext) region (Figure 4A). These results were found to be in line with the product description of Ventolin^®^ Diskus^®^ [21], where it was stated that 10 to 20% of the drug dose reaches the lower airways. Consequently, the in silico model for deposition was found adequate and used further to input SS pulmonary fractions into the PCAT module of GP.

For the PBPK model development in GP, the properties of SS listed in Table 1 were used. Once the physicochemical parameters were input into the model, the pharmacokinetic parameters of the drug were adjusted (i.e., clearance, AUC and t_max_). We first started by adjusting the renal clearance of the compound considering its excretion in urine 30 min after delivery (unchanged SS is secreted during this time period). Literature indicates that in relation to its maximum plasma concentration (C_max_), about 3.09 to 4.05 times more salbutamol can be found in urine during this time [12]. A renal clearance value of 4.2 mL/min/kg was found to be in line with the aforementioned mass ratio of salbutamol in urine. Although, no data could be found following administration via inhalation route, Cubitt et al. reported that for salbutamol the values of renal clearance after i.v. and oral administration were 4.8 and 4.3 mL/min/kg, respectively. Likewise, the used value for the renal clearance (4.2 mL/min/kg) was assumed to be realistic and left as such [13]. The rest of the compound clearance was assumed to be through the liver, following, the product description [21]. A hepatic clearance of 5.44 mL/min/kg was determined to fit best to the observed data and found to be in line with the results reported after i.v. and oral administration, 4.7 and 8.1 mL/min/kg, respectively [13]. In agreement with literature data, the total plasma clearance (9.62 mL/min/kg) was determined to be 43% renal and 57% hepatic [13]. In the PCAT module, the solubility of SS in simulated lung fluid (determined to be 384.70 ± 85.88 mg/mL by our working group [22]) was used as the pulmonary solubility of the compound. The apparent permeability of SS across Calu-3 and A549 cell lines described to be 8.53 × 10^−7^ ± 1.10 × 10^−7^ cm/s and 1.59 × 10^−5^ ± 0.04 × 10^−5^ cm/s [22], respectively, were used as input for the bronchiolar and alveolar compartments, respectively. The amounts of SS unbound to the cells of the alveolar and extra-thoracic compartments were optimized to 15% and 20%, respectively. Additionally, the fractions of swallowed and expectorated SS were also optimized. From the amount of SS deposited in the extra-thoracic region, it was considered that 65% of it was swallowed and 30% expectorated. Although, no literature information could be found to verify the optimized values for SS binding to cells and expectoration, the swallowed fraction was determined to be line with values reported in literature [23]. All the other remaining parameters in the PCAT module were left as default. The statistical summary of the developed PBPK model can be found in Tables S1 and S2 of the Supplementary Material, and the comparison of the plasma concentration profiles in vivo to the ones in silico can be found in Figure 4B.

### 3.5. In Silico Prediction of Salbutamol Sulphate Delivery to the Lung Using DPI Blends Containing Distinct Carriers 

**Figure 5:**
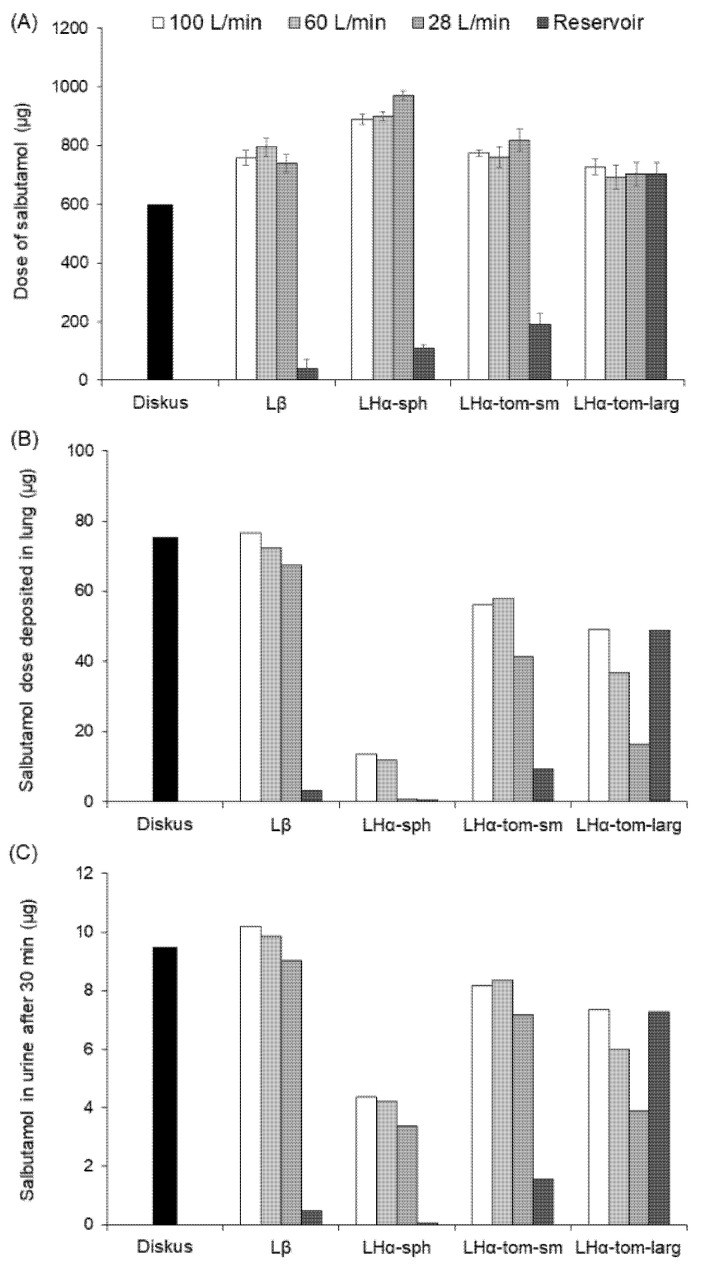
Comparison of the different carrier formulations delivered from distinct devices, taking Diskus^®^ Ventolin^®^ as a control for SS (**A**) emitted dose (*n* = 3, mean ± SD) and its predicted (**B**) mass deposited in the lung and (**C**) concentration in the urine.

Based on the aerodynamic profiles of SS determined from the NGI performance in vitro, the lung deposition of SS as well as its plasma concentration profiles were predicted in silico. To evaluate the performance of the different formulations, the delivered dose, lung deposition and the concentrations of non-metabolized salbutamol in urine after 30 min were evaluated (Figure 5) [24]. It was possible to observe that the dose was barely impacted by the airflow; however, a notably lower mass of API was emitted from the inhaler when the reservoir was used, with the exception of the LHα-tom-larg where no such effect was observed. The dose of SS arriving to the lung and the mass of non-metabolized salbutamol in the urine 30 min after administration followed similar trends. This confirmed that the latter is a good descriptor to evaluate the relatively bioavailability of salbutamol in the lung. Consequently, it was observed that a decrease in the airflow resulted in less salbutamol being available in the lung, and this was particularly evident for the LHα-tom-larg blend. The Lβ + SS blend was less affected, and for the LHα-tom-sm + SS mixture, only notable smaller doses were seen at 28 L/min. Compared to the other carriers, the blends of the API with LHα-sph led to a remarkably smaller dose of salbutamol arriving to the lung. For the reservoir device, only the blends containing LHα-tom-larg were able to generate salbutamol concentrations comparable to Cyclohaler^®^.

In Table 6, the PK parameters of the distinct salbutamol blends were compared with the ones from Diskus^®^ [7]. At all the airflows, the blends containing Lβ + SS and LHα-tom-sm + SS at 60 L/min and 100 L/min produced an identical C_max_ to Diskus^®^. In the case of the reservoir device, only LHα-tom-larg showed a C_max_ value approximate to Diskus^®^. All carrier blends showed values a t_max_ comparable to Diskus^®^, with the exception of LHα-sph + SS at 28 L/min. The latter showed a larger t_max_ value when in comparison with Diskus^®^. The predicted AUC_0–12h_ for the Lβ + SS blends was larger than that of the Diskus^®^ at 60 L/min and 100 L/min in the capsule-based device. At 28 L/min, the AUC_0–12h_ of the Lβ + SS blend was identical to the ones found for Diskus^®^. The LHα-tom-sm + SS blend showed to have similar AUC_0–12h_ to Diskus^®^ at all the tested airflows in the capsule-based device. For the LHα-sph + SS blend, the predicted AUC_0–12h_ at all the airflows in the Cyclohaler^®^ were slightly below the ones of Diskus^®^. None of the smaller carriers showed similar AUC_0–12h_ values to Diskus^®^ when used with the reservoir device. For LHα-tom-larg blend, the predict AUC_0–12h_ values of SS were similar to Diskus^®^, when the blend was tested at 100 L/min using de capsule-based device and at 60 L/min using the reservoir one.

### 3.6. Statistical Evaluation of the DPI Performance

**Figure 6:**
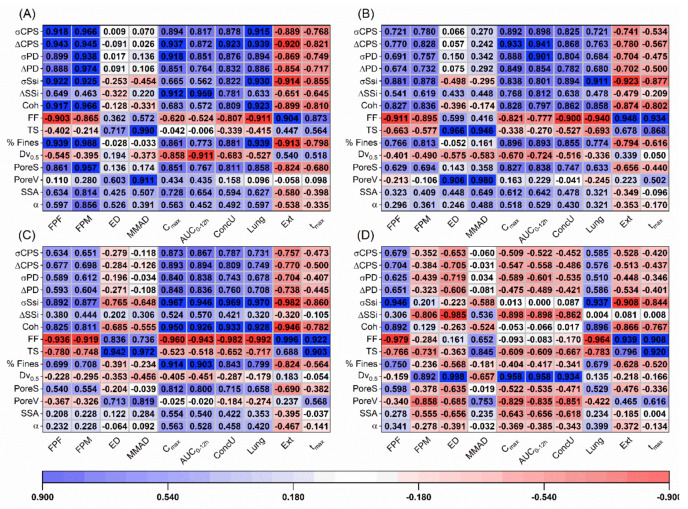
Pearson correlation coefficients between raw carrier properties and the in vitro–in silico performance of the capsule-based device at (**A**) 28 L/min, (**B**) 60 L/min as well as (**C**) 100 L/min and of the (**D**) reservoir inhaler (particle properties and respective nomenclature can be found in the supplementary material Table S3).

**Figure 7:**
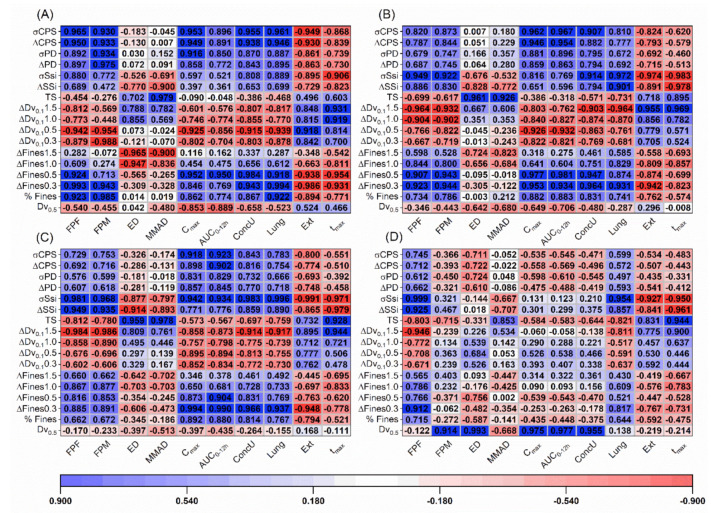
Pearson correlation coefficients between blend properties and the in vitro–in silico performance of the capsule-based device at (**A**) 28 L/min, (**B**) 60 L/min as well as (**C**) 100 L/min and of the (**D**) reservoir inhaler (bulk properties and respective nomenclature can be found in the supplementary material Table S3).

The variance of the particle and powder properties was compared, and no differences (*p* < 0.05) were found, when either the carriers (*p* = 0.477) or blends (*p* = 0.444) were tested. In turn, the influences of the different carriers (Figure 6) and blends properties (Figure 7) on the aerodynamic and PK parameters was evaluated using the Pearson correlation coefficient. It was observed that for both devices, aerosolization descriptors such as the FPF, Lung and Ext fractions as well as the t_max_ were fairly correlated with shear cell measurements, i.e., FF, σSSi and Coh. For the capsule device, the compression (σCPS and ΔCPS) and air permeability parameters (σPD and ΔPD) as well as the percentage of fines (%Fines) and the pore size (PoreS) correlated with the C_max_ and AUC_0–12_. Generally, the compression and permeability parameters showed a better correlation to the C_max_ and AUC_0–12_. The ConcU and FPM could be partially explained by the shear cell, compression and permeability measurements as well as the %Fines and the PoreS. For the capsule device, the FPM and FPF presented identical levels of correlation with the different carrier properties; for the reservoir inhaler, this was not the case. For the reservoir, the FPM, ED, AUC_0–12h_, C_max_ and ConcU were primarily described by the carrier mean particle size (Dv_0.5_). For the capsule-based device, no single parameter could explain the ED across all the tested airflows. At 100 and 60 L/min, the MMAD and ED could be correlated to the TS of the carriers. At 28 L/min, no clear parameter could be correlated to the ED, but the MMAD could still be explained by the TS. For the reservoir device, the TS was also somewhat related to the MMAD.

In case of the blends, the σSSi correlated well with the FPF, Lung and Ext fractions as well as the t_max_ for both devices. However, in this case, it was also observed that pressure titration descriptors, namely, the change in Dv_0.1_ (ΔDv_0.1_) and fines (ΔFines), were also able to correlate well with the aerosolization descriptors: FPF, Lung and Ext fractions; with the Dv_0.1_ showing a higher level of correlation. In case of reservoir device and the Cyclohaler^®^ (at 60 and 100 L/min), the change of Dv_0.1_ from 0 to 1.5 bar (ΔDv_0.1_1.5) was able to describe the aerosolization. For the capsule-based device used at 28 L/min, the change of Dv_0.1_ from 0 to 0.5 bar (ΔDv_0.1_0.5) correlated better to the aerosolization. Similar to the observations of the carrier powders alone, for the blends also the compression and pressured air permeability measurements as well as the %Fines (and also the change in the fines fractions from pressure titration) could generally be correlated with the C_max_, AUC_0–12h_ and ConcU. At 100 L/min, the change in fines from 0 to 0.3 bar (ΔFines0.3) was the parameter to better explain the C_max_, AUC_0–12h_ and ConcU. At 60 L/min, the change in fines from 0 to 0.3 bar and 0 to 0.5 bar (ΔFines0.5) could equally explain the aforementioned parameters. Lastly, at 28 L/min, the ΔFines0.5 was the one to better correlate to the PK parameters. For the reservoir device, equally to the results observed for carriers alone, the Dv_0.5_ was the parameter to better explain the FPM, PK parameters and ED. For the capsule device, also similar trends for the ED and MMAD to when the carriers were tested alone were found.

## 4. Discussion 

### 4.1. Particle Properties and Bulk Characteristics of the Different Lactose Grades 

Lβ was the carrier with the broader PSD due to the presence of a notable fraction of fine lactose particles, with a size of about 6 µm (Dv_0.1_ diameter, Figure 2). Due to its broader PSD, Lβ presented an irregular nature, higher surface asperity and cohesivity. This impacted the flowability of the carrier resulting in a lower FF, higher ΔSSi, ΔCPS and ΔPD, characteristic of more cohesive powders with poorer flowability [25,26,27].

LHα-sph showed the lowest cohesion tendency and the best flowability (lowest FF value). This was not surprising considering the spherical shape of its particles. It is known that when compared to more elongated shapes, spherical particles present reduced contact with surfaces leading to improved flowability [26,28,29]. Consequently, in comparison with the lactose powders of similar size, the spherical carrier presented the lowest ΔSSi, ΔCPS and ΔPD values. Compared with the other lactose grades, LHα-sph presented the highest values of TS, potentially, due to the fact that spherical particles are able to pack better, leading to a greater surface contact area during compaction [28,30]. Additionally, its rough surface might have further improved inter-particle contact (by particle interlocking) improving cohesion and leading to higher values of TS [30,31].

Between the carriers with the same size (Dv0.5 ≈ 50 µm), LHα-tom-sm presented intermediate values of FF; higher ΔSSi, ΔCPS and ΔPD and the lowest TS. The lower TS can be in one hand attributed to its tomahawk shape particles not being able to pack so efficiently and on the other hand to its smoother surface not having as many asperities able promote a more efficient particle cohesion and stronger bonding strength. Naturally, in relation to its larger counterpart (LHα-tom-larg), LHα-tom-sm presented slightly poorer flowability as translated by its lower FF and higher ΔSSi, ΔCPS and ΔPD values. The LHα-tom-sm also presented higher TS values in relation to LHα-tom-larg. These observations are in line with the well-established knowledge that, due to their larger surface area, smaller particles tend to be more cohesive; other works in literature show a similar trend between lactose grades differing in their particle size [26].

### 4.2. The Effect of the Force Distribution Balance on Salbutamol Plasma Concentration Profiles

De Boer et al. [32] have proposed the application of a force distribution concept (FDC) to understand drug attachment-detachment balance in carrier based DPI formulations. The FDC postulates that to understand DPI performance, one has to consider the forces that drive drug attachment during mixing and the ones that induce detachment during fluidization [32]. Considering that only the capsule device presented similar EDs for all the samples, we will discuss the detachment efficiency of the different carrier blends taking into consideration solely, the aerodynamic performance results of Cyclohaler^®^. The higher FPF of Lβ encountered at all flow rates clearly shows that compared to other carrier blends, the balance between attachment and detachment forces in the blends of this lactose grade leads to a more efficient aerosolization resulting in higher plasma concentrations of salbutamol. In relation to the attachment during mixing, the lower flowability of Lβ could have resulted in less efficient press-on forces during blending, leading to poorer API adhesion [33]. More specifically, the lower flowability due to the presence of a higher fraction of fines could have impacted mixing by (1) promoting the formation of agglomerates with the API, yielding SS-Lβ-fines composites that due to their larger size adhered more weakly to the coarser Lβ particles [34], and (2) creating small carrier surface asperities (lowest pore size between the small carriers) that could have resulted in weaker adhesion of the API to the Lβ surface [35,36]. Concerning detachment, the higher cohesion of the powder could have shifted the fluidization mechanism, leading to a more efficient aerosolization [25] or the formation of larger drug-excipient fines agglomerate could have led to the generation of greater inertial removal forces, beneficially impacting fluidization [33,34]. Finally, the presence of surface asperities could have reduced adhesion, facilitating detachment [35,36,37]. Independent of the mechanism(s) in action, it is hypothesized that the better performance showed by the Lβ blend was due to the presence of a certain amount of excipient fines.

From the carriers with smaller size, the lactose grade with the best flowability (LHα-sph) generated the highest press-on forces during mixing, promoting a stronger API attachment [33]. This in combination with the presence of large porosities on the LHα-sph surface allowed for multiple contact points with SS promoting its firm attachment. During aerosolization, the large pores were also able to provide shelter from the friction and drag forces, detrimentally impacting the performance [33,35]. Likewise, the blends of SS with LHα-sph resulted in very small predicted plasma concentrations of salbutamol.

For the LHα-tom-sm, its smoother surface and relatively good flowability could adequately promote the attachment of the SS to its surface. During aerosolization, the smoother nature of these particles resulted in a better exposure of the drug to the fluidization forces, resulting in relatively good predicted masses of SS arriving to the lung [33,38]. Similar to other works in literature, when comparing the two smoother carriers with distinct Dv_0.5_, it was observed that in comparison to its smaller counter-part, the LHα-tom-larg presented smaller predicted plasma concentrations of salbutamol [38] and was the lactose grade with the smaller delivered dose (further reducing the mass of SS arriving to the lung). This, might have been due to the fact that carrier particles with a larger size result in greater press-on on forces during mixing [34].

### 4.3. The Impact of Different Inhalers and Airflows on the Fluidization and Dose of Salbutamol Arriving to the Lung 

Differing aerosolization mechanisms, airflow resistances and dosing/dispersion systems are known to impact the masses of API arriving to the lung [39,40,41,42]. Considering this, the performance of the diverse DPI carriers was compared using two inhalers with distinct aerosolization mechanisms and sensitivities to airflow. Cyclohaler^®^ is a well-known capsule-based low-resistance device, in which the air passes through the powder formulation and the resulting mass of API arriving to the lung is dependent on the flow rate [39]. The chosen reservoir inhaler is a high to medium-resistance device, in which the aerosolization depends on cyclones/vortices that act on the carrier, promoting its impaction against the inhaler walls, and the mass of API arriving to the lung is independent from the airflow. Due to its dependency on the airflow, the Cyclohaler^®^ was tested using different flow rates and compared to the reservoir device at 60 L/min. The reservoir device and Cyclohaler^®^ showed comparable performances only when using the blend containing the LHα-tom-larg. It is hypothesized this was the case, because to promote fluidization the reservoir device relies on centrifugal forces and the capsule-based inhaler depends on the turbulent kinetic energy (TKE) of the airflow [39,43,44].

Centrifugal forces are known to act primarily on larger particles as their magnitude is proportional to the third power of their diameter [32]. Therefore, in order adequately fluidize a formulation, a minimum carrier size must be necessary when using devices based on centrifugal forces. Under these circumstances, the formulations composed of lactose grades having a Dv_0.5_ smaller than 130 µm showed not to be compatible with the reservoir device. In contrast, the high airflow resistance of the Cyclohaler^®^ able to effectively transfer the TKE into fluid dynamic shearing and mechanical impaction due to inertial forces showed to be compatible with all the tested carriers. The magnitude of the TKE is directly correlated to the airflow. The increase in TKE results in a greater number of impaction events per particle, improving API detachment and particle de-agglomeration [44]. Thus, a higher interdependency was observed between the Cyclohaler^®^ and the different airflows, resulting in distinct trends when the various lactose grades were used in combination with low loads of SS.

The Lβ + SS blends demonstrated the lowest dependency in relation to the used airflows, resulting in similar predicted salbutamol plasma concentration profiles and ConcU at 28, 60 and 100 L/min. The observation that the presence of a higher fraction of fines (Lβ has the higher fraction of particles <10 µm) might facilitate detachment, improving the performance, was in line with other findings in literature where similar observations were made at different flow rates [45]. This findings might have been related to (1) the weaker adhesion of SS to Lβ surface (explained in the previous section), leading to lower fluidization forces being necessary to induce detachment; (2) the API-excipient fines agglomerates, resulting in greater inertial separation forces (due to their larger size) that improve drug detachment from the carrier surface [46]; and (3) the presence of fines shifting the fluidization mechanism, where the cohesive powder is lifted as a plug and aerosolized more efficiently (in capsule devices) [25]. The MMAD of the Lβ + SS blends demonstrated a notably higher diameter in relation to the Dv_0.5_ of SS (1.53 µm) at all the analyzed flow rates. This indicated that some API detachment occurred in the form of particulate agglomerates and their breakage becomes less efficient as the airflow decreased (increase of the MMAD). Likewise, it seems evident that by one of the aforementioned mechanisms or a combination thereof, the presence of excipient fines facilitates detachment, resulting in relatively higher predicted masses of SS arriving to the lung even when lower airflow rates are used.

For LHα-sph, its very small predicted mass of SS arriving to the lung revealed that the fluidization forces used during aerosolization were not enough to adequately overcome API adhesion strength to the carrier surface. It is proposed that the high surface rugosity of the carrier leads to sheltering of the API from Cyclohaler^®^ shearing and the inertial forces, resulting only on larger API agglomerates being able to detach due to their higher inertia [33]. This was supported by the high MMAD found for SS when in combination with LHα-sph.

In comparison to Diskus^®^, the blends containing LHα-tom-sm were able to produce similar lung fractions of SS, when aerosolized at 100 and 60 L/min. A notable deterioration of the LHα-tom-sm performance could be observed when the airflow was lowered from 60 to 28 l/min. Direct comparison with its larger counterpart (LHα-tom-larg) revealed that the smaller lactose grade leads to larger predicted fractions of SS in the lung, demonstrating the superior API detachment efficiency of this blend. Comparison of SS MMAD evolution of the two carrier blends (LHα-tom-sm and LHα-tom-larg) showed that the bigger tomahawk carrier particles were hardly affected by the airflow, but the MMAD of the LHα-tom-sm blend was notably larger at 28 L/min. Based on these findings, it is proposed that the better aerosolization performance observed for the LHα-tom-sm blends might have resulted from a prevalence of fine particle agglomerates that are able to detach more easily from the excipient surface (due to their greater inertia) and can be more readily de-agglomerated at higher flow rates (explaining the higher MMADs found at 28 L/min). The presence of more agglomerates in the LHα-tom-sm mixture might have been the result of its higher fraction of fines in comparison to LHα-tom-larg [37,46] or simply due to its smaller Dv_0.5._ As explained in the previous section, smaller carriers generate lower press-on forces during mixing, so compared to its larger counter-part, the LHα-tom-sm might have been less efficient in breaking API agglomerates [34]. Likewise, by one of the aforementioned mechanisms or a combination thereof, it is proposed that the presence of more drug agglomerates in the LHα-tom-sm blend has a beneficial impact on its performance.

### 4.4. Implication of Carrier Properties on DPI Performance 

Now that it is understandable how excipient properties can impact delivery, it is important to analyze how particle and bulk characteristics descriptors can be practically used to interpret DPI performance. Within the context of this work (i.e., smaller sized carriers and low doses of SS), the susceptibility of a powder to an applied shear force, as represented by the shear cell parameters (FF, Coh and σSSi), can describe trends in the FPF and, consequent, predicted API deposition in Lung versus the Ext region and t_max_. The difference in the Dv_0.1_ obtained from the pressure titration experiments was also able to explain particle detachment fairly well relating to the FPF of the API and its predicted regional deposition and t_max_. It was also interesting to observe the correlation between the pressure used and the airflows. When higher airflows (i.e., 60 L/min and 100 L/min) were used, the difference in Dv_0.1_ from 0.1 bar to 1.5 bar was more relevant. At lower airflows (i.e., 28 L/min), the difference between 0.1 and 0.5 bar was the more important one. Likewise, it seems that powders that require higher shear forces to move resulted in higher FPF due to smaller press-on forces generated during mixing, resulting in lower API attachment, larger deposition on the lung and faster absorption of the drug into the bloodstream. The aforementioned observations were valid for both devices. However, none of the aforementioned measurements could completely explain the resulting performance in vivo as reflected by the correlations to the predicted C_max_, AUC_0–12_ and ConcU. It is suggested that this was due to the FPM not only being a result of the aerosolization efficiency (i.e., FPF) but also from the ED from the device. This was particularly clear in the reservoir device where it was observed that the mean size (Dv_0.5_) of the carrier particles was the critical parameter for the formulations correlating positively, with the ED, FPM, C_max_, AUC_0–12_ and ConcU. Only the blends containing Lα-tom-larg produced an adequate ED (≈2.7 mg of SS) and moderately good PK performance. In the case of the capsule-based device, all the powder blends produced an adequate ED in the 2.7–3.8 mg range. As a result, in the capsule-based device the FPM and FPF showed identical level of correlations, that is, the FPM also correlated well with the FF, Coh and σSSi of the raw carriers and the Dv_0.1_ obtained by pressure titration analysis of the blends. As the ConcU is a direct measurement of the bioavailability of SS in the lung, identical levels of correlations were found for this parameter and the FPM. For the capsule device, the C_max_ and AUC_0–12h_ correlated with compression (σCPS and ΔCPS) and air permeability parameters (σPD and ΔPD) of the raw carriers and the percentage of fines after pressure titration of the blends from 0.1 to 0.5 bar at 28 and 60 L/min (ΔFines0.5) and 0.1 to 0.3 bar at 100 L/min (ΔFines0.3). We hypothesize that the C_max_ and AUC_0–12h_ are not only a result of the mass of API arriving at the lower airways of the lung but also to the other parts of the respiratory system (i.e., upper airways) and as such dependent on all the aerodynamic particle size distribution (APSD) of the drug. Likewise, given that the APSD is a result of the interaction of the powder with the airflow, it is possible that the better correlation found for the CPS and PD parameter might have come from the fact that the values obtained from the measurements are correlated to the level of trapped air in the powder bed. For the ΔFines, as explained before, this is the fraction expected to arrive at the respiratory system and, naturally, correlated to the APSD and so also found to correlate with the C_max_ and AUC_0–12h_. The different interactions with the airflow, i.e., different correlations for the distinct flow rates, might be due to the fact that at 100 L/min, less fines are needed to impact detachment, so the presence of lower percentage of these (at lower PDPs less fines are detected when pressure increases from 0.1 to 0.3 bar) correlates better with performance. At lower airflows (28 and 60 L/min), it is suggested that fines play a more preeminent role in the detachment and the APSD of particles arriving to the respiratory tract, so the presence of a greater number of fines as higher pressures are used (from 0.1 to 0.5 bar) shows a better correlation with the C_max_ and AUC_0–12h_. Finally, the TS also seems to be of use for both the devices at all tested airflows. This parameter was able to provide an information on the attachment strength of the drug to itself and to possible carrier fine excipients, anticipating how the API would be aerosolized (single particles versus agglomerates) and thus the MMAD.

Although the presented results are only a reflection of in vivo performance generated from the simulations made in silico, and one can only speculate the in vivo response, it is apparent that for the capsule device, smaller carrier particles (Dv_0.5_ of about 50 μm) with a certain percentage of fines <10 μm (about 10%) are advantageous in producing a higher concentration of SS in the lung. The aforementioned performance in vivo was observed even at very low flow rates, which could reflect a scenario where patients are unable to produce enough inhalation forces (for example as a consequence of bronchospasm). For the reservoir device, the particle size (Dv_0.5_) of the carrier was identified as the critical parameter impacting the performance. However, none of the tested formulations led to a PK profile identical to Diskus^®^.

## 5. Conclusions

Through this work, we showcased the use of an in vitro–in silico approach supported by statistical analysis to derive mechanistic insights into the impact of particle and bulk carrier properties on the potential in vivo performance of DPIs. By applying this framework to carrier based DPI formulations containing low doses of SS with diverse lactose carrier types used in combination with capsule-based and reservoir devices, it was possible to understand that: (1) for the capsule device, smaller carrier particles (Dv_0.5_ of about 50 μm) with a certain percentage of fines <10 μm (about 10%) are advantageous in leading to higher concentrations of SS in the lung even at low flow rates; (2) for the reservoir device, particle size (Dv_0.5_) was the critical parameter identified to impact performance. To guarantee a formulation with the intended in vivo performance, the principle of operation of the inhaler should be first known and understood and carrier particle size chosen accordingly. Consequently, shear cell and permeability or compressibility measurements could be used to rank potential raw carrier powders between themselves. To anticipate detachment at different airflows and the MMAD, the particle size distribution by pressure titration and the tensile strength, respectively, could be applied.

## Figures and Tables

**Table 1:** Salbutamol physicochemical and pharmacokinetic parameters.

Parameter	Value	Source
Molecular weight (g/mol)	239.32	ADMET predicted
LogP (neutral species)	0.74	ADMET predicted
pK_a_	10.61 and 9.35	ADMET predicted
Reference solubility, pH= 7.4 (mg/mL)	270.00	Experimentally determined
Mean Precipitation Time (s)	900.00	Default
Diffusion coefficient (10^5^ cm^2^/s)	0.80	ADMET predicted
P_eff_ (10^4^ cm^2^/s)	1.33	ADMET predicted
Drug particle density (g/mL)	1.33	Experimentally determined
MMAD (µm)	1.83	Calculated based on Seheult et al. [8]
GSD (µm)	3.60	Calculated based on Seheult et al. [8]
Blood to plasma concentration ratio	0.96	Reported by Morgan et al. [11]
F_up_ (%)	92.00	Reported by Morgan et al. [11]
Mass ratio of urinary salbutamol (30 min) to maximum plasma concentration ^†^	3.09–4.05	Reported by Clark and Lipworth [12]
Renal Clearance (in relation to plasma clearance) (%)	26.00–57.00	Reported by Cubitt et al. [13]

^†^ Assuming a total plasma volume of 3.78 L [14]. P_eff_: effective permeability; MMAD: mean mass aerodynamic diameter; GSD: geometric standard deviation; F_up_: unbound fraction in plasma.

**Table 2:** Summary of the characteristics of the different lactose grades selected for this study.

Parameter	Lβ	LHα-sph	LHα-tom-sm	LHα-tom-larg	
Dv_0.5_ (µm) ^†^	52.89 ± 7.10	73.44 ± 2.96	60.26 ± 3.76	131.00 ± 1.32	
Fraction <10 µm (%) ^†^	13.48 ± 1.76	0.54 ± 0.45	3.34 ± 0.07	1.79 ± 0.03	
SSA (m^2^/g) ^¥^	0.51 ± 0.01	0.28 ± 0.00	0.15 ± 0.04	0.13 ± 0.01	
Pore volume (mm^3^/g) * ^¥^	1.20 ± 0.06	1.33 ± 0.17	0.53 ± 0.45	0.26 ± 0.03	
Pore size (nm) * ^¥^	11.52 ± 2.54	21.60 ± 2.54	17.61 ± 12.0	8.38 ± 0.65	
Shape Coefficient (α)	61.2	35.0	18.8	29.9	
Morphology	Irregular aggregates of anhydrous β-lactose	Rough, spherical agglomerates of α-lactose monohydrate	Smooth, tomahawk α-lactose monohydrate	Smooth, tomahawk α-lactose monohydrate	

Dv_0.5_: 50th percentile of the particle size distribution (volume based), SSA: specific surface area. * Pore size and volume by BET, only the porosities between 1.7 and 300 nm were determined. ^†^ *n* = 3, mean ± SD. ^¥^ *n* = 2, mean ± range.

**Table 3:** Powder parameters obtained from the FT4 evaluation of the different lactose grades (*n* = 3, mean ± SD).

Parameter	Lβ	LHα-sph	LHα-tom-sm	LHα-tom-larg
Coh	0.91 ± 0.13	0.30 ± 0.08	0.51 ± 0.12	0.57 ± 0.27
FF	7.51 ± 0.98	22.86 ± 6.16	14.25 ± 2.83	13.29 ± 5.09
σSSi (kPa)	6.00 ± 0.14	4.31 ± 0.14	5.08 ± 0.12	5.20 ± 0.12
ΔSSi (kPa)	4.98 ± 0.22	3.98 ± 0.16	4.82 ± 0.09	2.48 ± 0.14
σCPS (%)	23.44 ± 5.19	5.66 ± 0.32	8.62 ± 0.33	4.90 ± 0.83
ΔCPS (%)	15.46 ± 0.18	4.00 ± 0.22	7.10 ± 0.09	3.32 ± 0.13
σPD	4.72 ± 0.05	1.15 ± 0.03	1.67 ± 0.02	0.56 ± 0.04
ΔPD	2.93 ± 0.05	0.14 ± 0.00	0.36 ± 0.02	0.05 ± 0.00

Coh: cohesion, FF: flow function, SSi: incipient shear stress, CPS: change in volume after compression, PD: pressure drop across the powder bed. σ: mean of the SSi values between 5 and 15 kPa and CPS/PD values between 1 and 15 kPa; Δ: absolute difference between SSi at 5 and 15 kPa and CPS/PD values at 1 and 15 kPa.

**Table 4:** Powder rheological parameter obtained from FT4 evaluation of the different adhesive blends (*n* = 3, mean ± SD).

Parameter	Lβ + SS	LHα-sph + SS	LHα-tom-sm + SS	LHα-tom-larg + SS
σSSi (kPa)	6.27 ± 0.06	3.96 ± 0.09	5.55 ± 0.10	5.41 ± 0.41
ΔSSi (kPa)	5.14 ± 0.06	3.62 ± 0.03	5.31 ± 0.14	5.09 ± 0.14
σCPS (%)	19.07 ± 0.58	4.10 ± 0.26	9.59 ± 0.52	3.81 ± 0.35
ΔCPS (%)	14.16 ± 0.16	3.12 ± 0.09	6.56 ± 0.28	2.42 ± 0.14
σPD	7.37 ± 0.05	1.86 ± 0.00	2.57 ± 0.07	0.86 ± 0.11
ΔPD	4.61 ± 0.03	0.15 ± 0.01	0.60 ± 0.02	0.05 ± 0.00

SSi: incipient shear stress, CPS: change in volume after compression, PD: pressure drop across the powder bed. σ: mean of the SSi values between 5 and 15 kPa and CPS/PD values between 1 and 15 kPa; Δ: absolute difference between SSi at 5 and 15 kPa and CPS/PD values at 1 and 15 kPa.

**Table 5:** Summary of the in vitro aerodynamic performance (*n* = 3, mean ± SD).

Inhalation Settings	Blends	ED (mg)	FPM (mg)	FPF (%)	MMAD (µm)
Capsule-based 100 L/min	Lβ + SS	3.03 ± 0.10	0.80 ± 0.10	26.30 ± 2.56	2.66 ± 0.32
	LHα-sph + SS	3.56 ± 0.08	0.20 ± 0.06	5.66 ± 1.73	3.48 ± 0.77
	LHα-tom-sm + SS	3.10 ± 0.04	0.74 ± 0.02	23.85 ± 0.69	2.60 ± 0.13
	LHα-tom-larg + SS	2.91 ± 0.11	0.54 ± 0.01	18.68 ± 0.98	2.24 ± 0.16
Capsule-based 60 L/min	Lβ + SS	3.18 ± 0.12	0.72 ± 0.01	22.84 ± 1.01	3.28 ± 0.13
	LHα-sph + SS	3.60 ± 0.06	0.13 ± 0.02	3.74 ± 0.43	3.66 ± 0.12
	LHα-tom-sm + SS	3.04 ± 0.15	0.60 ± 0.04	20.03 ± 2.04	2.76 ± 0.26
	LHα-tom-larg + SS	2.77 ± 0.16	0.34 ± 0.02	12.53 ± 0.78	2.51 ± 0.08
Capsule-based 28 L/min	Lβ + SS	2.95 ± 0.33	0.62 ± 0.01	21.04 ± 2.14	4.62 ± 0.11
	LHα-sph + SS	3.89 ± 0.13	0.01 ± 0.00	0.24 ± 0.04	6.05 ± 0.51
	LHα-tom-sm + SS	3.27 ± 0.15	0.38 ± 0.01	11.62 ± 0.86	3.47 ± 0.03
	LHα-tom-larg + SS	2.81 ± 0.09	0.14 ± 0.01	5.04 ± 0.39	3.40 ± 0.05
Reservoir 60 L/min	Lβ + SS	0.40 ± 0.12	0.07 ± 0.04	17.60 ± 3.1	2.52 ± 0.12
	LHα-sph + SS	1.09 ± 0.08	0.02 ± 0.00	1.60 ± 0.11	1.09 ± 0.08
	LHα-tom-sm + SS	0.76 ± 0.14	0.10 ± 0.01	13.53 ± 0.84	0.76 ± 0.14
	LHα-tom-larg + SS	2.76 ± 0.11	0.33 ± 0.03	12.02 ± 0.68	2.75 ± 0.11

ED: emitted dose, FPM: fine particle mass, FPF: fine particle fraction, MMAD: mean aerodynamic diameter.

**Table 6:** Predicted pharmacokinetic parameters for salbutamol plasma concentration profiles after delivery using different carriers (predicted ± mean absolute error).

Carrier	Settings	C_max_ (10^−4^ μg/mL)	t_max_ (min)	AUC_0–12h_ (10^3^ μgh/mL)
Diskus^®^ *	Control	7.320 (6.48–8.270)	0.330 (0.17–0.50)	1.989 (1.790–2.209)
Lβ	28 L/min	7.011 ± 0.036	0.240 ± 0.067	2.168 ± 0.032
	60 L/min	7.657 ± 0.040	0.240 ± 0.067	2.345 ± 0.034
	100 L/min	7.854 ± 0.041	0.240 ± 0.067	2.341 ± 0.034
	Reservoir	0.360 ± 0.002	0.280 ± 0.078	0.113 ± 0.002
LHα-sph	28 L/min	4.510 ± 0.024	0.720 ± 0.201	1.620 ± 0.024
	60 L/min	4.569 ± 0.024	0.640 ± 0.179	1.656 ± 0.024
	100 L/min	4.593 ± 0.024	0.640 ± 0.179	1.665 ± 0.024
	Reservoir	0.520 ± 0.003	0.680 ± 0.190	0.188 ± 0.003
LHα-tom-sm	28 L/min	5.840 ± 0.030	0.320 ± 0.090	1.988 ± 0.029
	60 L/min	6.547 ± 0.034	0.280 ± 0.078	2.086 ± 0.030
	100 L/min	6.450 ± 0.034	0.280 ± 0.078	2.080 ± 0.030
	Reservoir	1.296 ± 0.007	0.333 ± 0.093	0.448 ± 0.006
LHα-tom-larg	28 L/min	3.716 ± 0.019	0.560 ± 0.157	1.346 ± 0.019
	60 L/min	4.888 ± 0.025	0.320 ± 0.090	1.667 ± 0.024
	100 L/min	5.826 ± 0.030	0.280 ± 0.078	1.901 ± 0.028
	Reservoir	5.729 ± 0.030	0.280 ± 0.078	1.853 ± 0.027

C_max_: maximum concentration observed, t_max_: time to reach maximum concentration observed, AUC_0–12_: area under the curve for the first 12 h. * From Moore et al. [7].

## Data Availability

The data presented in this study is available within the research and supplementary material of the article.

## Supplementary Materials

The following are available online at https://www.mdpi.com/1999-4923/13/3/297/s1. Table S1: Resume of the salbutamol PBPK statistical model. Table S2. Resume of the fitting of the pharmacokinetic parameters of salbutamol Table S3: Particle and bulk properties nomenclature used in the statistical analysis. Figure S1: Normalization of pressure titration results for the adhesive blends fine fraction (A) and 10th percentile of the particle size distribution (by volume) (B).
