# Supplementary material for: Understanding Carrier Performance in Low-Dose Dry Powder Inhalation: An In Vitro–In Silico Approach
Source: Pharmaceutics. 2021 Feb 24;13(3):297. doi: 10.3390/pharmaceutics13030297 (PMC8025906; doi:10.3390/pharmaceutics13030297)
Supplement: Supplementary file 1 [file pharmaceutics-13-00297-s001.zip › pharmaceutics-1113715-supplementary.pdf]

pharmaceutics

MDPI

Supplementary Materials: Understanding Carrier Performance in Low-Dose Dry Powder Inhalation: An In Vitro–In Silico Approach

Joana T. Pinto, Inês Cachola, João F. Pinto and Amrit Paudel

Table S1. Resume of the salbutamol PBPK statistical model.

Overall Fitting of the Model	
R^2	0.993
RMSE	0.023 ng/ml
MAPE	6.20%

RMSE: root mean square error, MAPE: Mean absolute prediction error.

Table S2. Resume of the fitting of the pharmacokinetic parameters of salbutamol.

Pharmacokinetic Parameters	MAPE
C_{max}	0.52%
t_{max}	28.00%
$AUC_{0\text{-}12h}$	4.99%
$AUC_{0\text{-}inf}$	1.46%

MAPE: Mean absolute prediction error, C_{max}: maximum concentration observed, t_{max}: time to reach maximum concentration observed, $AUC0_{\text{-}12}$: area under the curve for the first 12 h, $AUC_{0\text{-}inf}$: area under the curve to infinity.

Table S3. Particle and bulk properties nomenclature used in the statistical analysis.

Nomenclature	Definition
α	Shape coefficient
$Dv_{0.5}$	Diameter corresponding to 50% of the cumulative undersize of the volume distribution
%Fines	Percentage of fine particles < 10 µm (fines)
SSA	Specific surface area
PoreV	Pore volume
PoreS	Pore size
TS	Tensile strength
FF	Flow function
Coh	Cohesion
σSSi	Mean incipient shear stress between 5 and 15 kPa
ΔSSi	Incipient shear stress between difference between 5 and 15 kPa
σCPS	Mean change in volume after compression between 1 and 15 kPa
ΔCPS	Change in volume after compression difference between 5 and 15 kPa
σPD	Mean pressure drop values across the powder bed between 1 and 15 kPa
ΔPD	Difference between the normalized percentage of fines at 1 and 15 kPa
ΔFines0.3	Difference between the normalized percentage of fines at 0.1 and 0.3 bar
ΔFines0.5	Difference between the normalized percentage of fines at 0.1 and 0.5 bar
ΔFines1.0	Difference between the normalized percentage of fines at 0.1 and 1.0 bar
ΔFines1.5	Difference between the normalized percentage of fines at 0.1 and 1.5 bar
$\Delta Dv_{0.1}0.3$	Difference between the normalized size of the 10th percentile of the particle size distribution (by volume) at 0.1 and 0.3 bar
$\Delta Dv_{0.1}0.5$	Difference between the normalized size of the 10th percentile of the particle size distribution (by volume) at 0.1 and 0.5 bar
$\Delta Dv_{0.1}1.0$	Difference between the normalized size of the 10th percentile of the particle size distribution (by volume) at 0.1 and 1.0 bar
$\Delta Dv_{0.1}1.5$	Difference between the normalized size of the 10th percentile of the particle size distribution (by volume) at 0.1 and 1.5 bar
AUC_{0-12h}	Area under the curve for the first 12h
ConcU	Concentration in the urine after 30 min
C_{max}	Maximum concentration observed
ED	Emitted dose
Ext	Extra-thoracic region
FPF	Fine particle fraction
FPM	Fine particle mass
Lung	Lung region
MMAD	Mean mass aerodynamic diameter
t_{max}	Time to reach maximum concentration observed

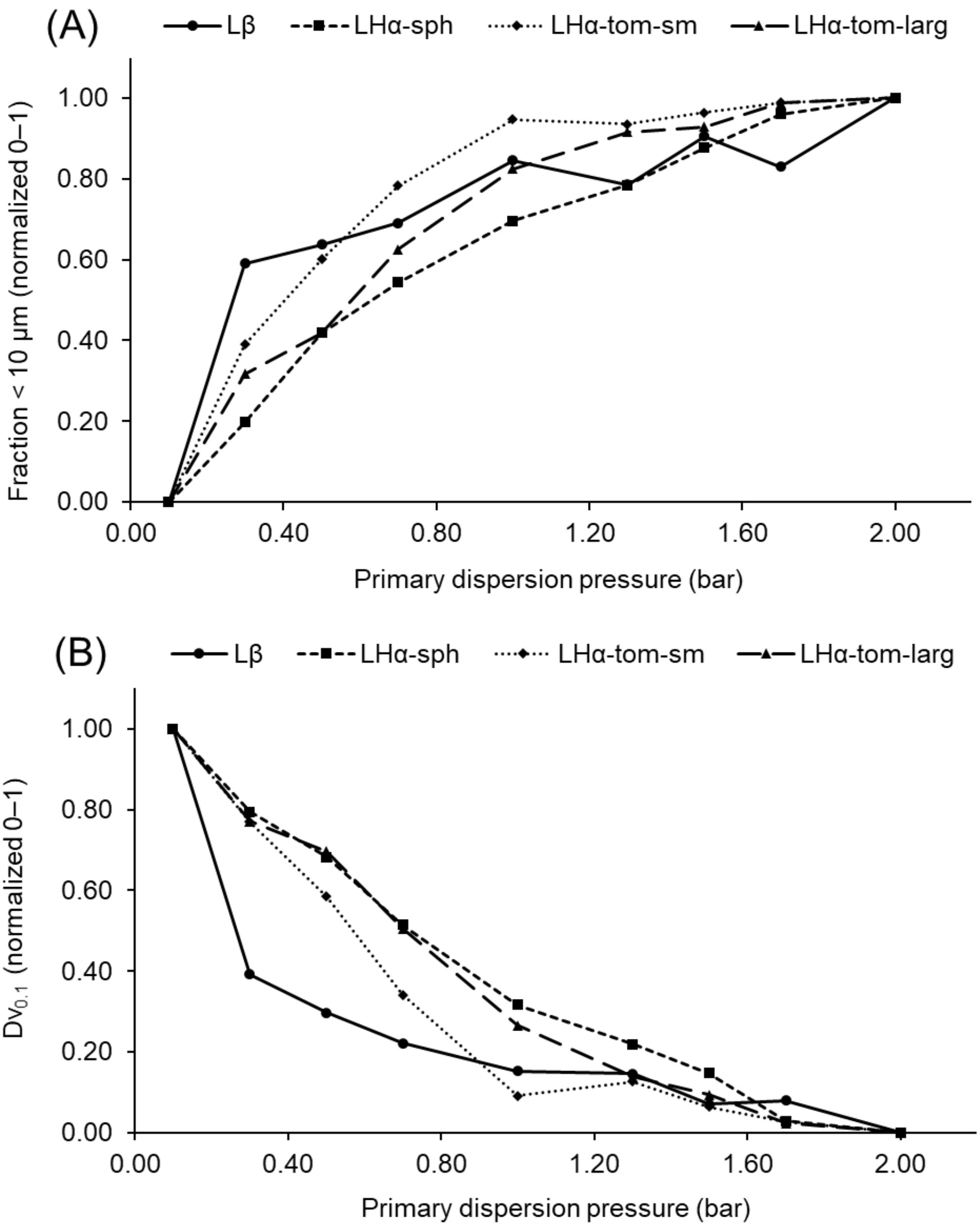

Figure S1. Normalization of pressure titration results for the adhesive blends fine fraction (**A**) and 10th percentile of the particle size distribution (by volume) (**B**).
